# XRK3F2 Inhibition of p62-ZZ Domain Signaling Rescues Myeloma-Induced GFI1-Driven Epigenetic Repression of the *Runx2* Gene in Pre-osteoblasts to Overcome Differentiation Suppression

**DOI:** 10.3389/fendo.2018.00344

**Published:** 2018-06-29

**Authors:** Juraj Adamik, Rebecca Silbermann, Silvia Marino, Quanhong Sun, Judith L. Anderson, Dan Zhou, Xiang-Qun Xie, G. David Roodman, Deborah L. Galson

**Affiliations:** ^1^Division of Hematology/Oncology, Department of Medicine, UPMC Hillman Cancer Center, The McGowan Institute for Regenerative Medicine, University of Pittsburgh, Pittsburgh, PA, United States; ^2^Division of Hematology-Oncology, Department of Medicine, Indiana University, Indianapolis, IN, United States; ^3^Hematology and Medical Oncology, Knight Cancer Institute, Oregon Health & Science University, Portland, OR, United States; ^4^Department of Pharmaceutical Sciences, School of Pharmacy, University of Pittsburgh, Pittsburgh, PA, United States; ^5^Richard L. Roudebush VA Medical Center, Indianapolis, IN, United States

**Keywords:** myeloma bone disease, p62-ZZ domain inhibitor, XRK3F2, GFI1, HDAC1, epigenetic, osteoblast suppression, chromatin immunoprecipitation

## Abstract

Multiple myeloma bone disease (MMBD) is characterized by non-healing lytic bone lesions that persist even after a patient has achieved a hematologic remission. We previously reported that p62 (sequestosome-1) in bone marrow stromal cells (BMSC) is critical for the formation of MM-induced signaling complexes that mediate OB suppression. Importantly, XRK3F2, an inhibitor of the p62-ZZ domain, blunted MM-induced *Runx2* suppression *in vitro*, and induced new bone formation and remodeling in the presence of tumor *in vivo*. Additionally, we reported that MM cells induce the formation of repressive chromatin on the *Runx2* gene in BMSC via direct binding of the transcriptional repressor GFI1, which recruits the histone modifiers, histone deacetylase 1 (HDAC1) and Enhancer of zeste homolog 2 (EZH2). In this study we investigated the mechanism by which blocking p62-ZZ domain-dependent signaling prevents MM-induced suppression of *Runx2* in BMSC. XRK3F2 prevented MM-induced upregulation of *Gfi1* and repression of the *Runx2* gene when present in MM-preOB co-cultures. We also show that p62-ZZ-domain blocking by XRK3F2 also prevented MM conditioned media and TNF plus IL7-mediated *Gfi1* mRNA upregulation and the concomitant *Runx2* repression, indicating that XRK3F2's prevention of p62-ZZ domain signaling within preOB is involved in the response. Chromatin immunoprecipitation (ChIP) analyses revealed that XRK3F2 decreased MM-induced GFI1 occupancy at the *Runx2-P1* promoter and prevented recruitment of HDAC1, thus preserving the transcriptionally permissive chromatin mark H3K9ac on *Runx2* and allowing osteogenic differentiation. Furthermore, treatment of MM-exposed preOB with XRK3F2 after MM removal decreased GFI1 enrichment at *Runx2-*P1 and rescued MM-induced suppression of *Runx2* mRNA and its downstream osteogenic gene targets together with increased osteogenic differentiation. Further, primary BMSC (hBMSC) from MM patients (MM-hBMSC) had little ability to increase H3K9ac on the *Runx2* promoter in osteogenic conditions when compared to hBMSC from healthy donors (HD). XRK3F2 treatment enriched *Runx2* gene H3K9ac levels in MM-hBMSC to the level observed in HD-hBMSC, but did not alter HD-hBMSC H3K9ac. Importantly, XRK3F2 treatment of long-term MM-hBMSC cultures rescued osteogenic differentiation and mineralization. Our data show that blocking p62-ZZ domain-dependent signaling with XRK3F2 can reverse epigenetic-based mechanisms of MM-induced *Runx2* suppression and promote osteogenic differentiation.

## Introduction

Multiple myeloma (MM) is the second most common hematologic malignancy and the most frequent cancer to involve bone (1, 2). Over 80% of patients develop osteolytic bone lesions that can result in severe bone pain, frequent pathological fractures and hasten mortality (3–5). MM patients with fractures have a 20% increased risk of death as compared to MM patients without fractures (4). Therefore, the clinical and economic impact of bone disease in patients with MM can be catastrophic. MM cells in the bone marrow microenvironment increase osteoclast (OCL) differentiation, which generates the bone lesions (6). Unfortunately, MM bone lesions rarely heal due to MM-induced alteration of osteoblast precursors (preOB) within the bone marrow stromal cell (BMSC) population that prevents their differentiation into bone-forming osteoblasts (OB) (7). In addition, the MM altered bone microenvironment enhances support of MM growth, survival, and drug-resistance (8). Importantly, the MM-induced OB suppression persists after eradication of MM cells, suggesting that MM cells induce repressive, heritable, epigenetic changes at the *Runx2* gene, the key transcription factor required for OB differentiation (9). Thus, new bone formation at the site of MM lytic lesions is suppressed or absent, resulting in lesions that persist after MM cells are eradicated (7). Although new therapies for MM that target both MM cells and the bone compartment have greatly improved progression-free survival and overall survival, most patients eventually develop resistance to the available treatments and MM remains an incurable disease (10). Further, although proteasome inhibitors have been reported to transiently increase bone formation in MM patients (11), a lack of anabolic bone agents that can reliably repair bone lesions in MM patients remains a major clinical challenge. Thus, studies that address the underlying pathophysiology of MM effects on the bone environment are critical to develop new approaches to improve the quality of life and enhance the survival of MM patients.

Increasing evidence demonstrates that BMSCs from MM patients display distinctive tumor-promoting features and impaired osteogenic differentiation as compared to normal donors (12). Several deregulated signaling molecules and receptor pathways, including the Wnt signaling inhibitor DKK1 (13), sclerostin (14), the cytokines IL3, IL7, TNFα (15, 16), and the chemokine cytokine ligand 3 (CCL3) (17), are associated with anti-osteogenic, pro-osteolytic and growth-supporting properties of the myeloma tumor-microenvironment. However, the mechanisms responsible for the prolonged propagation of osteogenic-inhibition of MM-BMSCs in the absence of persistent myeloma signals are still largely unresolved.

The autophagic cargo receptor and signaling platform protein p62 (sequestosome-1) is an important modulator of bone turnover, and mutations associated with its impaired function result in skeletal disorders such as Paget's disease of bone (18). As a scaffold protein, p62 is a multi-domain adaptor protein modulates and integrates signaling by interacting directly with signaling proteins from multiple cell surface receptors (e.g., TNFα-TNFR signaling mediated via the RIP1 binding domain of p62 (ZZ domain) and RANKL-RANK, IL1β-ILIR, NGF-TrkA mediated via the TRAF6 binding domain of p62), connecting them to multiple downstream pathways (e.g., NFκB, p38 MAPK, PKCζ, JNK) [for a review see (19)]. This multifunctional protein also serves as a scaffold molecule connecting proteasomal and autophagic protein degradation (20). Its elevated expression is also associated with increased resistance to proteasome inhibitors in MM (21, 22).

TNFα induces RIP1 interaction with the ZZ domain of p62. A study by Hiruma et al. (23) demonstrated that p62 is required for stromal cell support of MM growth and OCL formation (23). Both MM cell and TNFα required the presence of p62 in BMSC (23) for their induction of the protein levels of vascular cell adhesion molecule-1 (VCAM1), which mediates BMSC-MM cell interactions (24), IL6, a pro-inflammatory and myeloma pro-survival factor (25), and RANKL, important for osteoclastogenesis (26, 27). Importantly, the p62-ZZ domain was found through deletion analyses to be specifically required for these activities (28). We recently reported the identification of a novel small molecule inhibitor the p62-ZZ domain of signaling, XRK3F2, that blocks TNFα and MM activation of downstream signaling from the p62-signaling hub (29). In addition, XRK3F2 also directly decreased OCL formation. Further, XRK3F2 directly inhibited cell growth of primary CD138+ MM cells and human MM cell lines *in vitro*, without negatively affecting the growth of BMSC. However, XRK3F2 did not reduce MM growth in a 5TGM1-MM mouse model. Surprisingly, a periosteal reaction was observed in the tibiae directly injected with MM and treated with XRK3F2, but not in the contralateral non-MM-injected limb or saline-injected controls, indicating that XRK3F2 induced new cortical bone formation in the 5TGM1-murine model of Multiple myeloma bone disease (MMBD) *in vivo* (29).

We reported that BMSC from MM patients expressed elevated levels of the transcriptional repressor GFI1 at both the RNA and protein level (30). Similarly, GFI1 was elevated in murine BMSC exposed to MM *in vitro* or *in vivo*. Knock-down of GFI1 was found to decrease the ability of MM to induce OB suppression and could reverse established Runx2 repression (30). GFI1 is a transcriptional repressor of *Runx2* in BMSC that directly binds and recruits the chromatin corepressor complex consisting of HDAC1 and EZH2 to the *Runx2-P1* promoter (31). Enrichment of these histone modifiers inhibits transcriptional activity of *Runx2* by reducing the active chromatin mark acetylated histone H3 at lysine 9 (H3K9ac) and enhancing the repressive chromatin mark trimethylated H3 at lysine 27 (H3K27me3) at the *Runx2* promoter (31). This epigenetic-based mechanism maintains inhibition of the *Runx2-P1* promoter even in the absence of MM exposure, which results in a prolonged suppression of BMSC differentiation into OB. In a study by Wang et al. (32), downregulation of GFI1 in response to AMPK activation in MC4 preOB upregulated gene expression of the osteogenic mediator *Osteopontin* (*Opn*), which promoted osteogenesis. The molecular function of GFI1 has been primarily investigated during the differentiation of lymphoid and myeloid cells (33, 34), and there are only a few reports of its activity in osteogenic cells and very little is known about its transcriptional and post-translational regulation (35, 36). We tested the hypothesis that XRK3F2 might be generating new bone growth in MM-bearing bone by blocking GFI1 epigenetic repression of *Runx2*.

## Materials and methods

### Reagents

Cell culture media, penicillin and streptomycin (pen/strep), DTT, and all DNA primers were from Invitrogen. FCS was from Atlanta Biologicals (S12450). Ascorbic acid (A4403) was from Sigma-Aldrich. Histone 3 (H3) (9715) Ab was from Cell Signaling. Chromatin immunoprecipitation (ChIP) Abs for H3K9ac (61251) and HDAC1 (40967) were from Active Motif. GFI1 (ab21061) Ab was from Abcam. GoTaq Flexi DNA polymerase was from Promega. TRIzol reagent (10296028) was from Life Technologies. Mouse recombinant TNFα (410-MT) was from R&D Systems.

### Cell lines, primary murine BMSC, and co-cultures

All cultures described below contained 10% FCS-1% pen/strep. The pre-OB murine cell line MC3T3-E1 subclone-4 (MC4) was obtained from Dr. Guozhi Xiao (37, 38) in 2009 and subclone-14 (MC14) was obtained from ATCC (CRL-2594) in 2014. MC3T3-E1 subclone-4 (MC4) was used in experiment 1A and MC3T3-E1 subclone-14 (MC4) was used for the rest of the experiments. Both were maintained in ascorbic acid-free αMEM proliferation media. MM cell lines were generously provided by Dr. Steven Rosen (MM1.S) and Babtunde O Oyajobi (5TGM1) were maintained in RPMI1640. The stably transduced murine 5TGM1-GFP-TK (5TGM1) MM cells (30) and human MM1.S-GFP cells (23) were previously described. Cell lines were authenticated by morphology, gene expression profile, and tumorigenic capacity (MM cells). MC4 cells were grown to 90% confluency prior to co-culture. MM1.S Conditioned media was generated by growing MM1.S cells for 24 h at confluence of 1 × 10^6^ cells/ml. Harvested media was filtered using a 0.22-μm filter prior to its use in experiments. Direct 5TGM1-MC4 (10:1) co-cultures and indirect co-cultures of MM1.S cells in transwells (10:1) with MC4(14) cells were carried out in 50:50 RPMI1640/αMEM proliferation media. MM1.S in transwells (Corning Inc., 3450) or 5TGM1 cells were carefully removed (FACS analysis demonstrated that ≤ 1% 5TGM1 cells remained). The MC4 (14) cells were isolated immediately or subjected to OB differentiation first. BM cells were isolated from C57BL/6 mice femurs and tibia. Animal studies were approved by the IACUC at the VA Pittsburgh Healthcare System. BM cells were harvested from tibiae and femurs as previously described (30). After overnight incubation, the non-adherent cells were removed and the remaining stromal cell population was washed with PBS and maintained in ascorbic acid-free αMEM-10% FCS, 1% pen/strep proliferation media. BMSC were expanded for 2.5 weeks to reach optimal confluence. Co-cultures with MM cells or cytokine treatments and RNA preparation analyses were conducted as described for MC4 cells.

### Human samples and primary hBMSC cultures

BM aspirates were collected in heparin from 5 healthy donors and 7 MM patients. This study was carried out in accordance with the recommendations and protocol approvals by the University of Pittsburgh and Indiana University Institutional Review Boards (IRBs). All subjects gave written informed consent in accordance with the Declaration of Helsinki. BM mononuclear cells were separated by Ficoll-Hypaque density sedimentation and the nonadherent cells removed after overnight incubation in Iscove's Modified Dulbecco's Medium (IMDM)-10%FCS. The adherent cultures were then continued for 21 d with media changes every 4 d to obtain BMSC. Subconfluent cells were detached with trypsin and replated (10^5^ cells/10-cm dish) for use at passage 2 and 3.

### OB differentiation, and alkaline phosphatase and alizarin red assays

OB differentiation media (αMEM supplemented with 50 μg/ml ascorbic acid and 10 mM β-glycerophosphate, and 10 nM Dex) was added to primary hBMSC; media was changed every 3 days. Alkaline phosphatase staining was performed using SIGMAFAST BCIP/NBT (Sigma, B5655-5TAB) protocol. Mineralization at 20 days was assessed using alizarin red staining (30). The staining density quantitation was carried out using a ProteinSimple FluorChem™ M imaging system.

### Real-time quantitative PCR (*q*PCR) RNA expression analyses

RNA was isolated using TRIzol reagent and converted to cDNA using First-Strand cDNA Synthesis System (Life Technologies, 11904-018). *q*PCR was carried out using 2x Maxima SYBR Green/ROX *q*PCR Master Mix (K0223, Thermo Fisher) in Fast 96-Well Reaction Plates (Applied Biosystems) using a StepOnePlus (Applied Biosystems). Relative mRNA levels were calculated using the ΔΔCt method using *18SrRNA* for normalization. The *q*PCR primers are listed in Table 1.

**Table 1:** *q*PCR primers for Mouse (m) and Human (h) mRNA analysis.

**Gene**	**Forward primer (5′->3′)**	**Reverse primer (5′->3′)**
m*Runx2*	CCTCTGACTTCTGCCTCTGG	ATGAAATGCTTGGGAACTGC
m*Gfi1*	GGCTCCTACAAATGCATCAAATG	TGCCACAGATCTTACAGTCAAAG
m*18srRNA*	GAGCGACCAAAGGAACCATA	CGCTTCCTTACCTGGTTGAT
*mOCN*	TAGTGAACAGACTCCGGCGCTA	TGTAGGCGGTCTTCAAGCCAT
*mBSP*	AAGAAGAGGAAGAGGAAGAAAATGA	GCTTCTTCTCCGTTGTCTCC
*mOsx (Sp7)*	AGAGGTTCACTCGCTCTGACGA	TTGCTCAAGTGGTCGCTTCTG
*mIL6*	CAAAGCCAGAGTCCTTCAGA	GCCACTCCTTCTGTGACTCC
*mVcam1*	TGCCGAGCTAAATTACACATTG	CCTTGTGGAGGGATGTACAGA
*hRUNX2*	CATTTCAGATGATGACACTGCC	GTGAGGGATGAAATGCTTGG
*hGFI1*	GAGCCTGGAGCAGCACAAAG	GTGGATGACCTTTTGAAGCTCTTC

### Chip assays

Chromatin from MC4 cells, MM-BMSC, and HD-BMSC was analyzed using a modification of the ChIP Millipore/Upstate protocol (MCPROTO407) as described (31, 39) using Magna ChIP Protein A+G Beads (16-663, Millipore). In brief, a total of 2 × 10^7^ cells were fixed in 1% formaldehyde (F79-500, Fisher) for 10 min at room temperature. Samples were sonicated (to generate DNA fragments of 250 base pairs (bp) average length) on ice using a Fisher Scientific Sonic Dismembrator (Model 100) and centrifuged at 12,000 RPM for 10 min. Chromatin from 4 × 10^6^ cells was diluted 7-fold in ChIP Dilution Buffer (0.01% SDS, 1.1% Triton X-100, 1.2 mM EDTA, 16.7 mM Tris-HCl, pH8.1, 167 mM NaCl) and incubated at 4°C overnight with respective antibodies. Aliquots for input and non-specific IgG control samples were included with each experiment. IgG ChIP was run on untreated MC4 samples. ChIP-*q*PCR primers are listed in Table 2. Fold enrichment was calculated based on Ct as 2^(Δ*Ct*)^, where ΔCt = (Ct_Input_ – Ct_IP_). The IgG ΔCt was subtracted from the specific Ab ΔCt to generate ΔΔCt = (ΔCt_specificAb_ – ΔCt_IgG_).

**Table 2:** Murine and human ChIP-*q*PCR *Runx2-P1* primers.

ChIP amplicons^*^	**Forward (5′->3′)**	**Reverse (5′->3′)**
Murine −670	AAGGCAAACAGAAGGAAGCA	TGCTGCTTTGCAGTAATTCG
Murine −36 (3)	TGAGGTCACAAACCACATGA	TGAAGCATTCACACAATCCAA
Murine +150 (5)	CGTTTTGTTTTGTTTCCTTGC	CCCAGTCCCTGTTTTAGTTG
Murine +363 (6)	CAGGGACTGGGTATGGTTTG	ACGCCATAGTCCCTCCTTTT
Murine +33130	AGGTAGCCCAGCAAAAACCT	CCCCTCTGTGAGCCAAAATA
Human +185	CACCGAGACCAACAGAGTCA	TGGTAACATGTGAAAAGCAAAGA
Human +66065	AAGGCCCCACCTCTAACACT	AGACAACAGGCGAGGCTAAA

* *Numbers represent midpoints of amplicons relative to the Runx2-P1 transcription start site. Numbers in parentheses were used to designate the amplicons in our previous publication (31)*.

**Table 3:** Multiple myeloma patient characterization.

**ID**	**Age**	**Gender**	**Race**	**Newly diagnosed**	**ISS stage**	**Skeletal disease**
MM1	60	M	White	Yes	I	No
MM2	55	M	Unknown	No	II	Yes
MM3	76	M	White	No	Unknown	Yes
MM4	80	M	White	No	I	No
MM5	58	F	White	No	II	Yes
MM6	50	M	White	No	II	Yes
MM7	44	F	White	No	I	Unknown

### Statistical analysis

All experiments were repeated at least two independent times. Most data is presented as biological triplicates and results reported as means±SD unless otherwise stated. Statistical significance was evaluated by either the Student's *t*-test using Graphpad Prism 6 as indicated. Degree of significance is represented using ρ values: ^*^ρ ≤ 0.05, ^**^ρ ≤ 0.01, ^***^ρ ≤ 0.001, ^****^ρ ≤ 0.0001 (Different symbols may be used to reflect multiple two-way comparisons).

## Results

### XRK3F2 prevents and reverses MM-induced *Gfi1* upregulation and rescues OB gene expression in MM suppressed preOB

**Figure 1:**
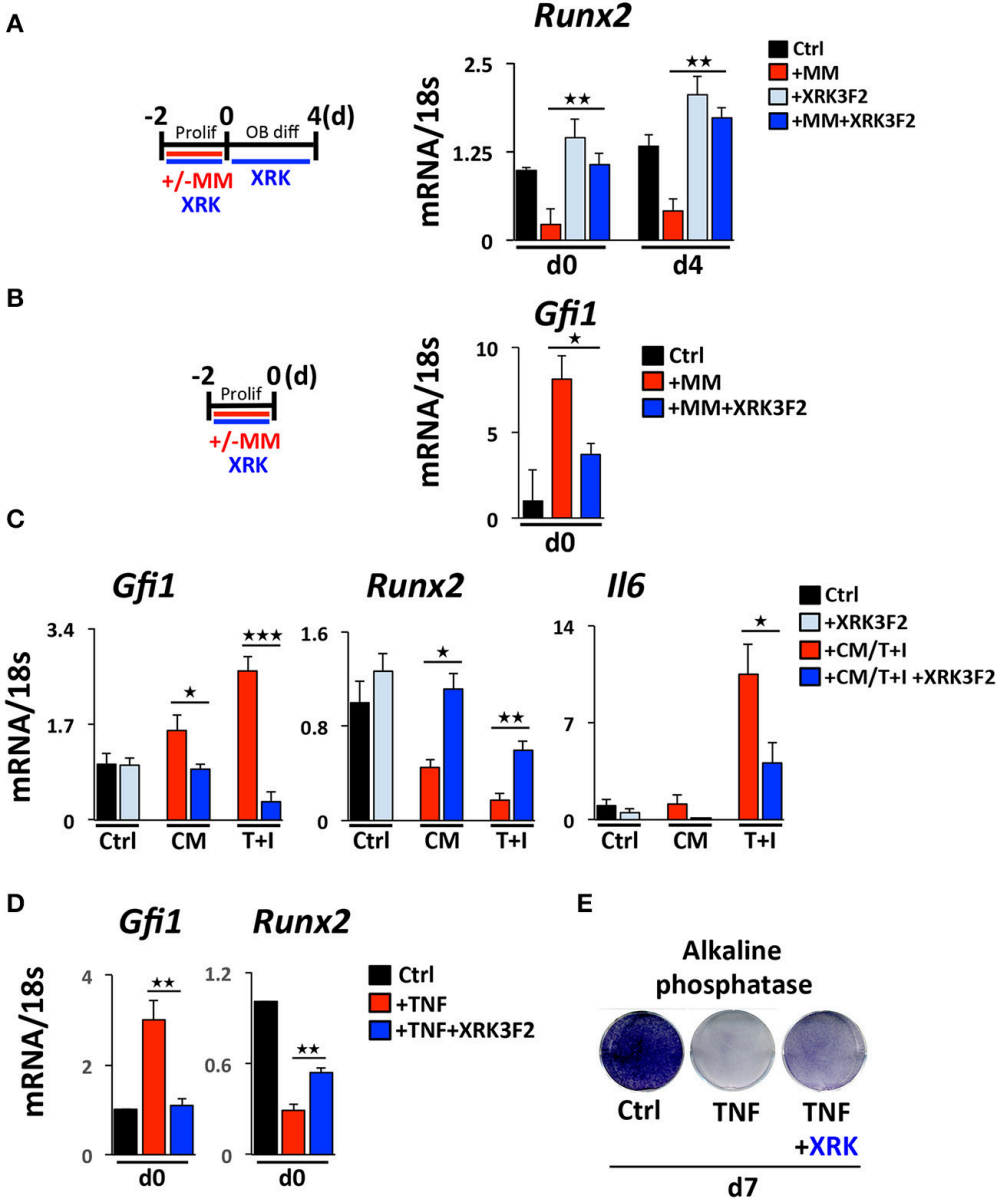
Upregulation of GFI1 in myeloma pre-OB is blocked by XRK3F2. **(A)** As depicted in the schematic, MC4 cells were cultured in with or without 5TGM1 MM cells (in direct contact) for 48 h under proliferation conditions +/– XRK3F2 (5 μM). MM cells were then removed by washing and media was changed to osteogenic differentiation conditions +/– XRK3F2. MC4 cells were collected at the time of MM cell removal (day 0), and after 4 days of differentiation culture in the absence of MM cells (day 4). **(B)** MM cells were co-cultured with MC4 cells for 48 h under proliferation conditions +/– XRK3F2 (5 μM). **(C)** Primary murine BMSC were treated with MM1.S conditioned media (described in Materials and Methods) or TNF plus IL7 (5 ng each) for 48 h. **(A–C)** Expression levels of *Gfi1, Runx2* and *Il6* were measured using qPCR as indicated. SEM for 3 biological replicates is indicated. **(D)** MC4 cells were cultured with vehicle or TNFα (10 ng/ml) +/– XRK3F2 (3 μM) for 48 h under proliferation conditions. Expression levels of *Gfi1* and *Runx2* were measured using qPCR as indicated. SD for 3 biological replicates are indicated. **(E)** MC4 cells were cultured with or without TNFα (2.5 ng/ml) +/– XRK3F2 (3 μM) for 7 days under differentiation conditions. Results present alkaline phosphatase staining representative of 3 biological replicates. ^*^p ≤ 0.05; ^**^p ≤ 0.01; ^***^p ≤ 0.001.

While little is known about how MM cells upregulate GFI1 in preOB, we have previously reported and demonstrate in this study that both TNFα and IL-7 can upregulate *Gfi1* mRNA and induce its nuclear translocation in MC4 preOB (30, 31). We investigated if p62 signaling plays a role in MM cell upregulation of GFI1 expression and induces GFI1-mediated epigenetic repression of *Runx2*. Direct co-culture (48 h) of murine 5TGM1 MM with murine preOB MC4 cells in proliferation media suppressed *Runx2* mRNA (Figure 1A, d0). The *Runx2* mRNA inhibition persisted for 4 days after removal of MM cells and addition of osteogenic media (Figure 1A, d4). The presence of XRK3F2 during MM-preOB co-cultures prevented *Runx2* suppression at both d0 and d4 (Figure 1). Furthermore, XRK3F2 blocked MM-induced upregulation of *Gfi1* (Figure 1B). To determine if XRK3F2 directly affects the preOB response to MM signals in MM-preOB co-cultures, we determined if XRK3F2 could block the ability of MM1.S conditioned media or a combination of TNFα plus IL7 to induce *Gfi1* expression in primary mouse BMSC. XRK3F2 blocked the induction of *Gfi1* mRNA in BMSC in both treatment conditions (Figure 1C). In contrast, XRK3F2 prevented both MM1.S CM and TNFα plus IL7-mediated *Runx2* suppression. Further, the pro-inflammatory and myeloma pro-survival factor *IL6* mRNA was also reduced by XRK3F2 treatment (Figure 1C). In addition, XRK3F2 also prevented TNFα-mediated upregulation of *Gfi1* and rescued inhibition of *Runx2* in MC4 preOB (Figure 1D). The prevention of TNFα-induced suppression of preOB by XRK3F2 was further confirmed by increased levels of alkaline phosphatase staining in preOB (Figure 1E). This suggests that a direct XRK3F2-mediated inhibition of p62 signaling within preOB prevents *Gfi1* induction by MM signaling, which prevents GFI1 suppression of *Runx2* in BMSC.

### XRK3F2 prevents and reverses epigenetic suppression of *Runx2* by blocking the recruitment of GFI1 and its co-repressor HDAC1 to the *Runx2-p1* promoter

**Figure 2:**
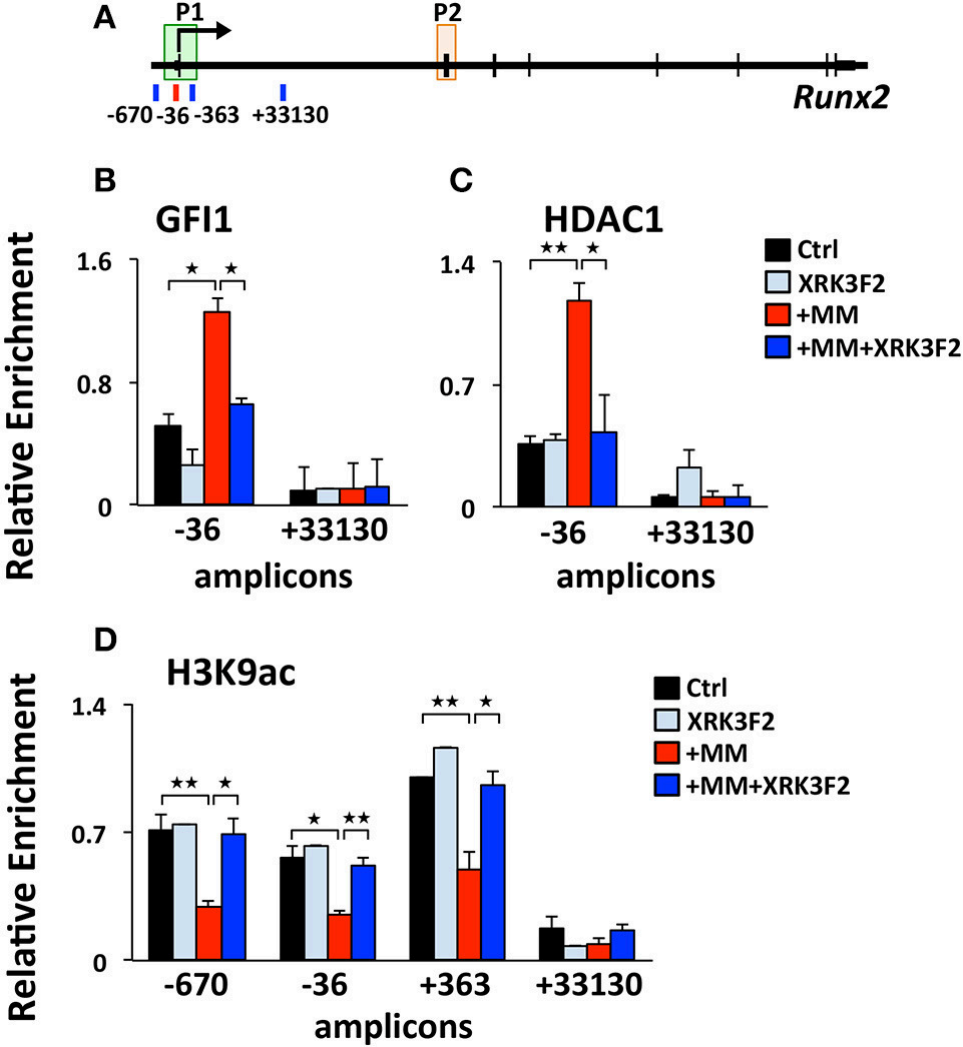
XRK3F2 prevents MM-induced decrease of H3K9ac at the *Runx2* promoter by blocking the recruitment of GFI1 and histone deacetylase HDAC1. **(A)** Schematic representation of the murine *Runx2* gene and positions of amplicons used for ChIP-qPCR analysis. Shown are ChIP data for **(B)** GFI1, **(C)** HDAC1 binding and **(D)** H3K9ac levels at the *Runx2-P1* promoter in MC4 preOB after 48 h in proliferation media control (d0), control treated with 5 μM XRK3F2 (d0+XRK3F2), 5TGM1-MM-treated (d0+MM), and 5TGM1-MM-treated in the presence of XRK3F2 (d0+MM+XRK3F2). IgG non-specific control was subtracted in the H3K9ac data set. SEM represents 2 (for GFI1 and HDAC1) and 3 (for H3K9ac) biological replicates. ^*^p ≤ 0.05; ^**^p ≤ 0.01.

We previously reported that MM cells induce the transcriptional repressor Gfi1 to directly bind to the *Runx2-P1* promoter in preOB cells and recruit the chromatin corepressor HDAC1 to *Runx2*, reducing euchromatin marks such as H3K9ac (30, 31). Importantly, this reduction persists in the absence of MM cells, suggesting that these epigenetic changes result in long term OB suppression. Therefore, we tested if XRK3F2 prevents the GFI1-mediated epigenetic suppression of *Runx2* observed following MM exposure using ChIP-qPCR analysis of the murine *Runx2-P1* promoter using the amplicons depicted (Figure 2A). In MC4 preOB, XRK3F2 prevented MM-induced GFI1 occupancy at the *Runx2-P1* promoter (Figure 2B) and recruitment of the chromatin co-repressor HDAC1 (Figure 2C). Consistent with the lack of HDAC1 recruitment, histone acetylation levels of H3K9 at *Runx2-P1* were not reduced in XRK3F2-treated MM-exposed preOB (Figure 2D). As a control, we also evaluated the H3K9ac status at the center of the long intron between the two *Runx2* promoters where GFI1 does not bind, and observed that HDAC1 is not recruited there, and MM exposure did not modify the H3K9ac status. This data argues that XRK3F2 can prevent the MM induced recruitment of the GFI1-HDAC1 complex to the *Runx2-P1* promoter, thus blocking establishment of the repressive chromatin architecture at the *Runx2* gene and, thereby, protecting the capacity for OB differentiation.

### XRK3F2 rescues transcriptional suppression of *Runx2* by reversing the recruitment of the GFI1-HDAC1 complex to the *Runx2-P1* promoter

**Figure 3:**
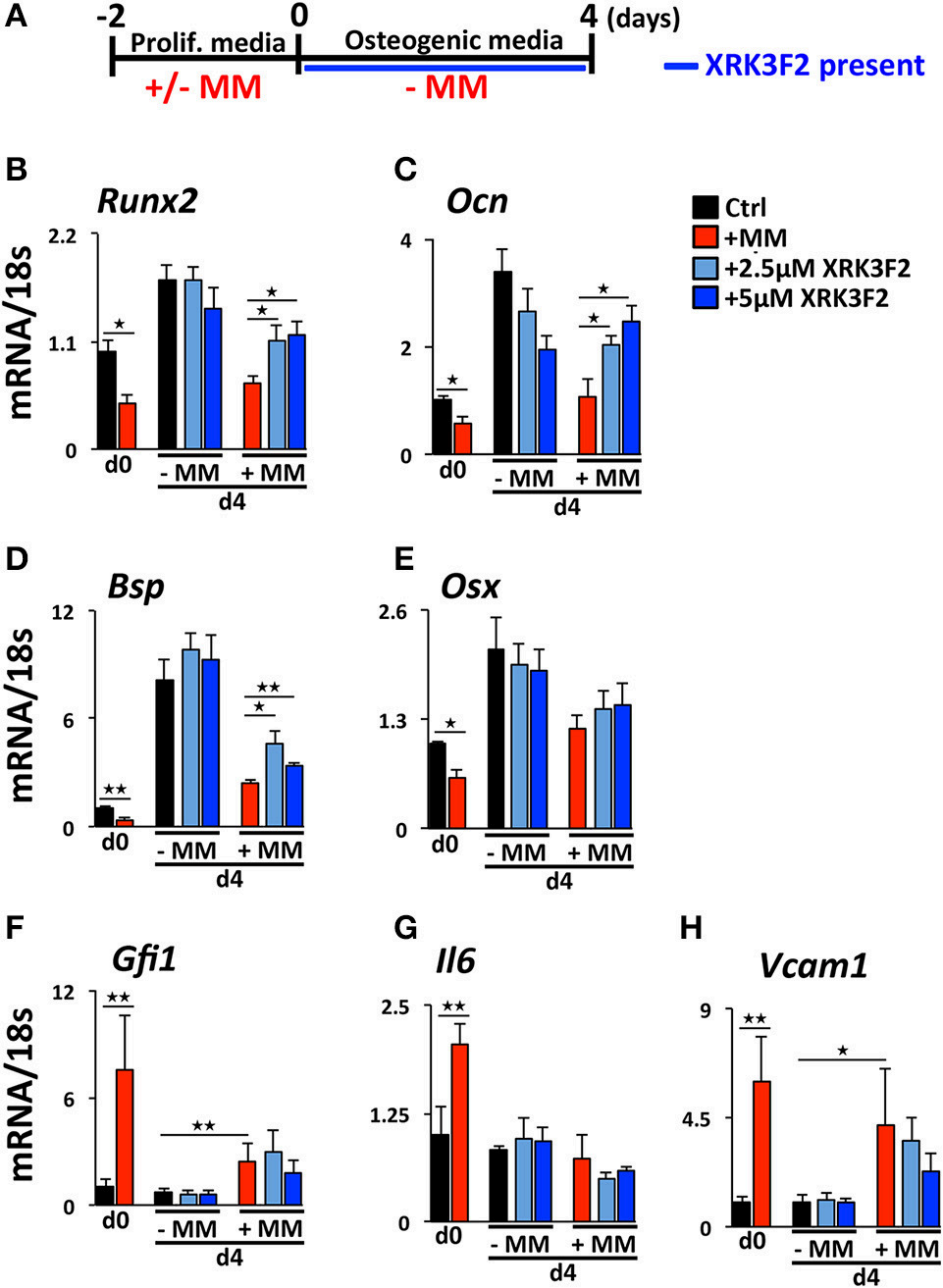
XRK3F2 rescues expression of osteogenic genes in MM-exposed preOB. **(A)** As depicted in the experimental design schematic, MC4 cells were cultured with or without 5TGM1 MM cells (in a direct contact) for 48 h under proliferation conditions. MM cells were then removed by washing (d0) and media was changed to osteogenic conditions +/– XRK3F2 (2.5, 5 μM) for 4 days (d4). MC4 cells were harvested at both d0 and d4 for mRNA analyses. qPCR mRNA profiles for **(B)** *Runx2*, **(C)** *Ocn*, **(D)** *Bsp*, **(E)** *Osx*, **(F)** *Gfi1*, **(G)** *Il6*, and **(H)** *Vcam1* are shown. SEM for 3 experimental wells and a representative of 2 biological replicates are shown. ^*^p ≤ 0.05; ^**^p ≤ 0.01.

**Figure 4:**
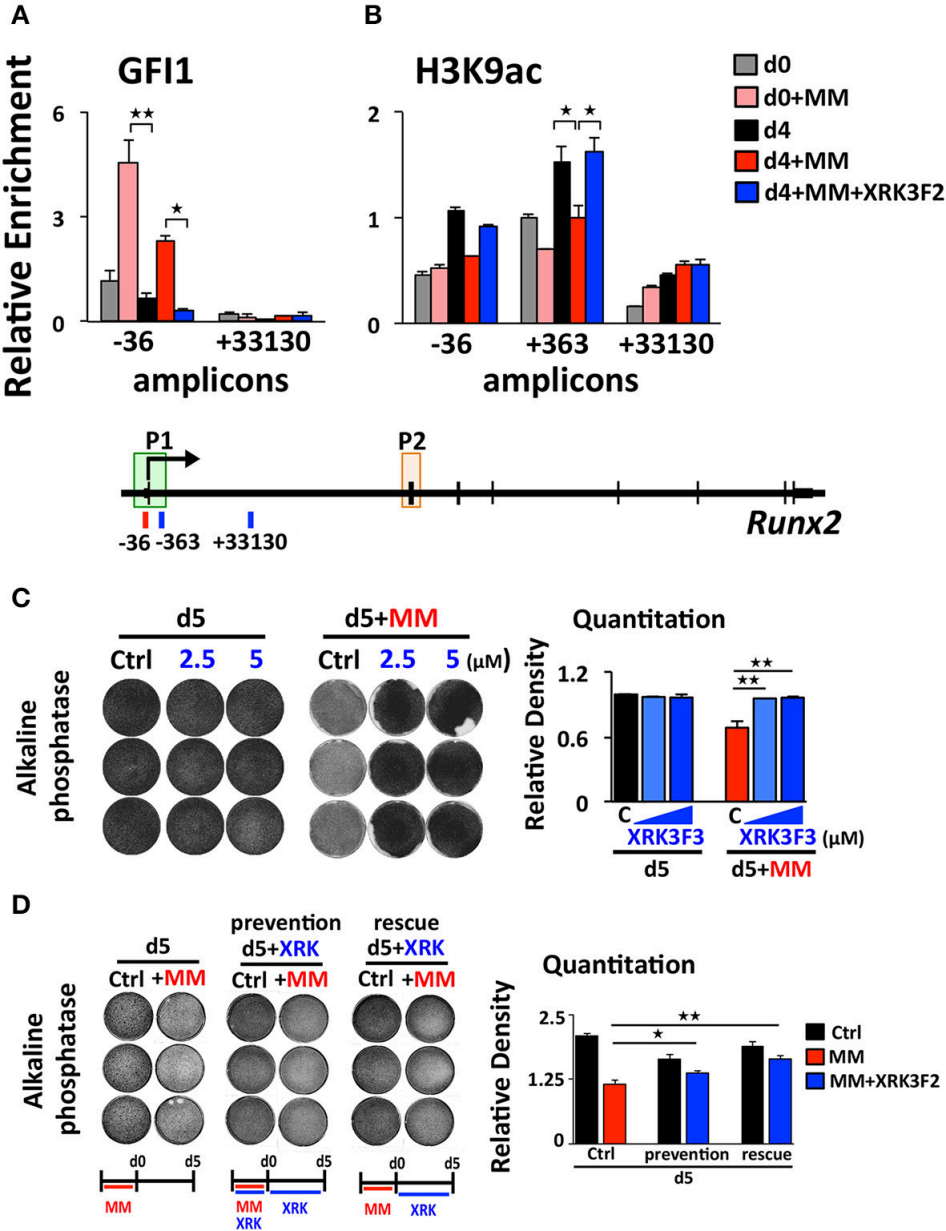
XRK3F2 reverses GFI1 occupancy and reverses loss of H3K9ac at *Runx2* in MM-exposed preOB. **(A)** MC4 cells were cultured as depicted and described in 3A, with XRK3F2 added only after MM cells were removed. Shown are ChIP data for **(A)** GFI1 occupancy and **(B)** H3K9ac at the *Runx2-P1* promoter obtained from MC4 cells harvested after culture (48 h) in proliferation media in the absence or presence of 5TMG1 cells in direct co-culture (d0, d0+MM) or continued in the absence of MM cells in osteogenic media (d4, d4+MM) or continued in osteogenic media with 5 μM XRK3F2 (d4+MM+XRK3F2). **(C)** MC4 preOB were co-cultured with MM1.S (direct contact) for 72 h in proliferation media, MM cells were removed and remaining preOB were subjected to osteogenic differentiation for 5 days +/– XRK3F2 (2.5, 5 μM). **(D)** MC4 preOB were co-cultured with MM1.S (in transwells) for 72 h in proliferation media +/– 5 μM XRK3F2 (during), then the MM cells were removed and the preOB were subjected to osteogenic differentiation for 5 days +/– 5 μM XRK3F2 (after). MC4 were treated with XRK3F2 either during MM exposure or afterwards, but not both. **(C,D)** Alkaline phosphatase staining with quantitation measurements is shown as a representative of 2 independent experiments. SEM for 3 experimental wells and representative of 2 biological replicates is indicated. ^*^p ≤ 0.05; ^**^p ≤ 0.01.

We reported that maintenance of the MM-induced Runx2 suppression in the absence of MM cells requires the continued presence of GFI1 and HDAC1 activity (30, 31). Therefore, we performed a set of “rescue” experiments to test whether XRK3F2 can be used to reverse the epigenetic suppression of preOB following MM exposure. In this model, MC4 preOB were co-cultured in direct contact with 5TGM1 MM cells in proliferation media. After 48 h, the MM cells were removed and the MM-exposed MC4 cells were subjected to osteogenic differentiation in the presence or absence of 2 doses of XRK3F2 (Figure 3A). Addition of either dose of XRK3F2 to differentiating MM-exposed preOBs significantly elevated *Runx2* mRNA together with downstream RUNX2 target genes Osteocalcin (*Ocn)*, Bone sialoprotein (*Bsp)* and Osterix (*Osx)* (40), which are critical for osteogenic differentiation (Figures 3B–E). However, genes induced by MM, including *Gfi1, Il6*, and *Vcam1*, which we have shown are sensitive to XRK3F2 inhibition during preOB MM or TNFα exposure [Figure 1C and (23, 29)], did not respond to XRK3F2 after the MM cells were removed (Figures 3F–H). The MM-induced expression of *Gfi1* mRNA after 48 h (d0) was reduced after MM cell removal, but was persistently expressed at a low level in MM-exposed MC4 during 4 days of osteogenic differentiation as compared to preOB not exposed to MM. We did not observe a significant difference in *Gfi1* mRNA with XRK3F2 treatment in day 4 differentiated preOBs (Figure 3F). ChIP analyses demonstrated that enhanced binding of GFI1 at the *Runx2-P1* promoter persists 4 days following MM removal (Figure 4A). In the XRK3F2 “rescue treatment” paradigm, in which XRK3F2 was added to MC4 preOB osteogenic cultures after 5TGM1 MM cells (direct contact) were removed, the amount of GFI1 binding at the *Runx2-P1* promoter in MM-exposed MC4 preOB was significantly reduced while the levels of H3K9ac increased (Figure 4B). This XRK3F2 rescue treatment also restored OB differentiation as reflected in alkaline phosphatase staining (Figure 4C). In a similar experiment, XRK3F2 was used in both prevention (present during co-cultures) and rescue (added after MM cell removal) models in a transwell experiment using MM1.S and MC4 preOB. Alkaline phosphatase activity was quantified after 5 days of differentiation in osteogenic media. Consistent with the previous results, alkaline phosphatase staining showed that XRK3F2 rescued osteogenesis of preOB exposed to MM cells indirectly in trans-wells (Figure 4D). These results are consistent with the observations that even after MM-exposure, XRK3F2 decreased GFI1 binding and rescued chromatin acetylation at the *Runx2-P1* promoter, resulting in elevated *Runx2* expression.

### XRK3F2 rescues acetylation levels at the *Runx2* promoter in MM patient hBMSC

**Figure 5:**
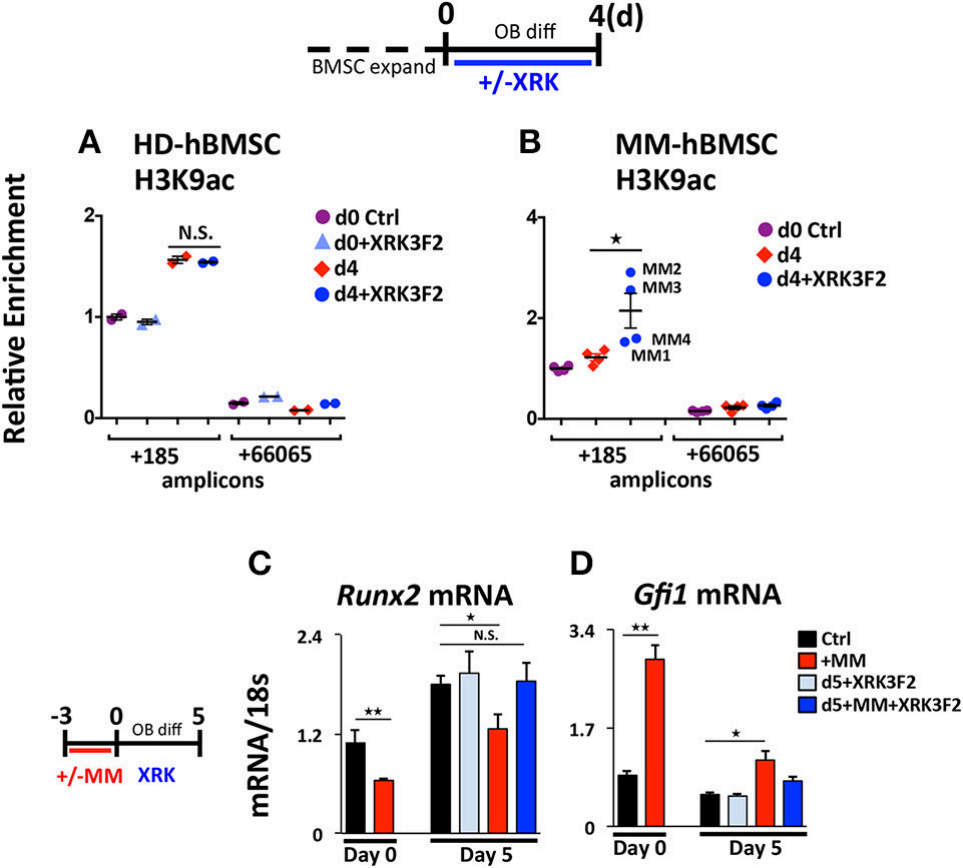
XRK3F2 rescues H3K9ac levels and subsequent osteogenic differentiation of MM hBMSCs. Human BMSC samples were expanded in proliferation media and harvested for ChIP-*q*PCR at either: (Day 0) the day of the switch into osteogenic media +/– XRK3F4 (5 μM) or (Day 4) after four days of differentiation. In the control healthy donor (HD)-BMSC, the d0+XRK3F2 sample was kept in proliferation media from d0 through d4 in the presence of XRK3F2. Anti-H3K9Ac ChIP-*q*PCR analysis of **(A)** HD-hBMSC (*n* = 2) and **(B)** MM-hBMSC (MM) (*n* = 4, MM patient samples MM1-4, Table 3) using amplicons +185 and +66065 relative to the *hRunx2* P1 TSS. Day 0 (d0) anti-H3K9Ac ChIP amplicon +185 N sample result was used as the reference sample for other samples on graphs and ΔΔCt shown. **(C)** Healthy donor BMSC were co-cultured with MM1.S for 72 h in proliferation media, MM cells were removed and the remaining hBMSC were subjected to osteogenic differentiation for 5 days +/– XRK3F2 (5 μM). qPCR profiles for *Runx2* **(C)** and *Gfi1* **(D)** mRNA are shown. SEM for 3 experimental wells and representative of 2 biological replicates is indicated. ^*^p ≤ 0.05; ^**^p ≤ 0.01.

We tested the ability of XRK3F2 to reverse the MM-induced long-term repressive chromatin architecture on the *Runx2* gene after MM exposure *in vivo*. We compared the effects of XRK3F2 on the *Runx2* promoter acetylation levels during differentiation of healthy normal donor (HD-hBMSC) and MM patient hBMSC (MM-hBMSC). As Figure 5A demonstrates, the H3K9 acetylation levels at *Runx2* increased when the HD-hBMSC were cultured for 4 days in osteogenic media as a result of activation of osteoblast differentiation pathways (31, 41). XRK3F2 did not affect the increase in *Runx2* promoter H3K9ac levels during normal differentiation. In contrast, the H3K9ac levels at *Runx2* remained unresponsive to osteogenic signals in MM patient hBMSC (Figure 5B). However, XRK3F2 treatment significantly rescued the H3K9ac levels at *Runx2* in MM patient hBMSC, which suggested that XRK3F2 would enhance their response to osteogenic differentiation. Therefore, we set up co-cultures of primary HD-hBMSC with the MM1.S MM cell line in hBMSC proliferation media (Figures 5C,D). After removal of the MM cells, we subjected the MM-exposed hBMSC cells to osteogenic differentiation for 5 days in the presence of vehicle or XRK3F2. As Figure 5C demonstrates, MM1.S cell exposure prevented the *Runx2* increase after osteogenic stimuli, which is consistent with chromatin repression of the *Runx2* promoter. Addition of XRK3F2 following MM1.S cell removal and addition of osteogenic media rescued the *Runx2* mRNA levels, consistent with the results obtained with mouse cells in Figure 3B. Further, MM cell co-culture increased *Gfi1* expression in the HD-hBMSC, which persisted for 5 days after MM cell removal (Figure 5D). XRK3F2 addition after MM removal decreased *Gfi1* mRNA, although the difference did not reach significance. These observations are consistent with the observation that the GFI1-HDAC1 complex is required to both establish repression and to persistently repress *Runx2* in BMSCs in the absence of MM cells. Further, these data reveal that signaling through the p62-ZZ domain is required in the absence of MM cells, suggesting the induction of feed-forward suppressive autocrine signaling.

### XRK3F2 rescues the OB mineralization potential of MM patient hBMSC

**Figure 6:**
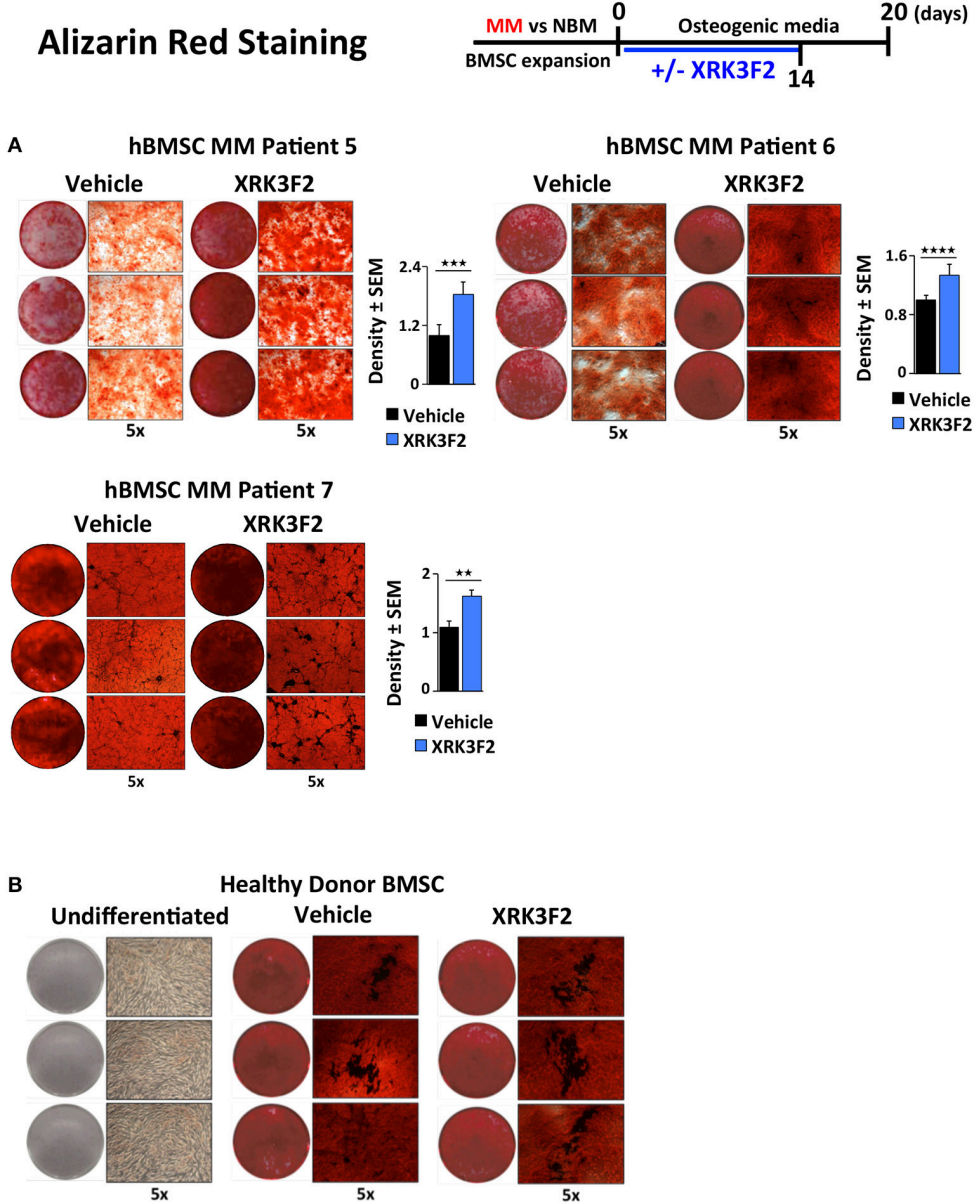
XRK3F2 rescues MM-BM-MSC OB mineralization. **(A)** Myeloma (MM)-BMSC from three different patients (MM patient samples MM5-7, Table 3) were cultured for 20 days in osteogenic media. Vehicle control (DMSO) or XRK3F2 (5 μM) was supplemented only for the first 14 days of the differentiation experiment. The presence of calcium deposition/mineralization was assessed using Alizarin red staining. Three independent wells from each treatment group per patient are shown, with 5X magnification images next to entire wells. However, 6 wells from each patient sample group were used for quantitation of Alizarin red staining density using the ProteinSimple AlphaView software, with SEM indicated. **(B)** Alizarin red images of undifferentiated HD-BMSC (negative control) and HD-hBMSC treated with vehicle (DMSO) or XRK3F2 (5 μM) during incubation in osteogenic media for 20 days. ^**^p ≤ 0.01; ^***^p ≤ 0.001; ^****^p ≤ 0.0001.

Since BMSCs obtained from MM patients exhibit an impaired ability to differentiate into mineralizing OB, and XRK3F2 can rescue early steps in osteogenesis, we asked whether XRK3F2 could rescue the complete osteogenesis pathway as demonstrated by the ability to mineralize. We cultured MM-hBMSC for 20 days in the presence of vehicle or XRK3F2 (through day 14) in osteogenic media and assessed their mineral deposition using Alizarin Red staining. Addition of XRK3F2 significantly increased mineralization by the MM-hBMSC from 3 patient samples (Figure 6A) as compared to the vehicle control. Figure 6B shows that 5 μM XRK3F2 did not affect differentiation of HD-BMSC.

## Discussion

**Figure 7:**
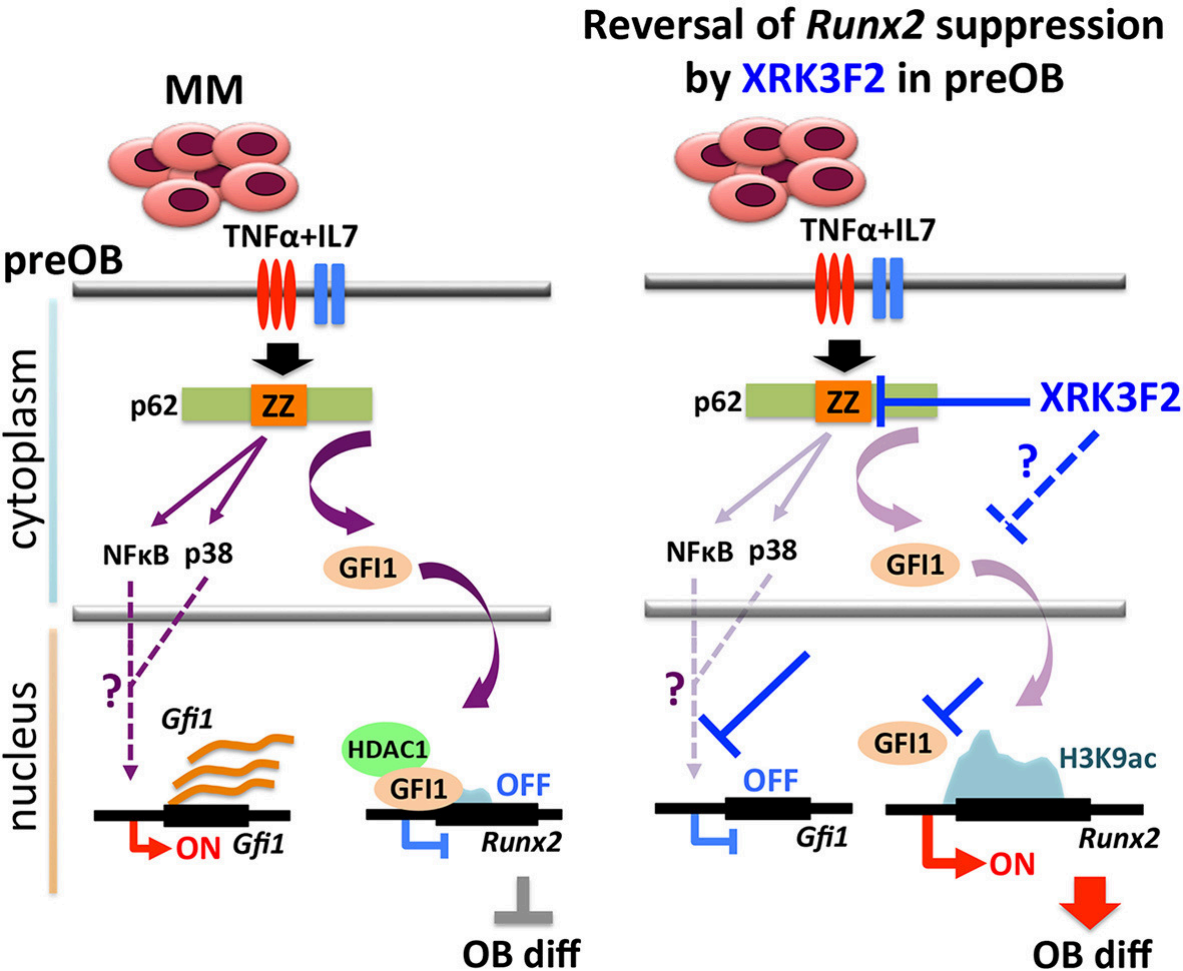
Proposed mechanism of XRK3F2 drug action on p62 signaling in MMBD. MM cell co-culture or TNFα plus IL7 stimulation of preOB activate p62-ZZ domain signaling, which results in activation of downstream pathways involving NFκB and p38 MAPK. Further, p62-ZZ domain activation increases GFI1 levels, which subsequently translocates into the nucleus, binds the *Runx2* gene and recruits the chromatin modifier HDAC1 to deacetylate and repress the *Runx2-P1* promoter. Inhibition of the p62-ZZ domain by XRK3F2 may act in different ways to prevent transcriptional repression of *Runx2* by GFI1. First, by suppressing activation of transcription factors such as NFκB and/or C/*EBP*β, thus preventing *Gfi1* transcription. Second, by inhibiting nuclear translocation of GFI1, thereby preventing its ability to target the *Runx2* promoter. In both scenarios XRK3F2 prevents GFI1 from instigating epigenetic suppression of *Runx2*, which allows for subsequent progression of osteoblastogenesis. Further, XRK3F2 blocks GFI1 maintenance of the epigenetic repression in the absence of MM, thereby allowing its reversal and rescuing the osteoblastogenesis potential.

In this paper, we address the mechanisms associated with XRK3F2-mediated *Runx2* derepression in myeloma-exposed preOB. The involvement of MM-induced GFI1-mediated epigenetic suppression of *Runx2* expression in BMSC prompted us to examine whether p62 signaling is associated with the GFI1-*Runx2* inhibition axis (30, 31). First, we recapitulated our previous findings in which MM exposure upregulated *Gfi1* mRNA and protein expression in BMSC from MM patients and MM-injected mice (30). Blocking p62 signaling using XRK3F2, the p62-ZZ domain inhibitor, prevented GFI1 upregulation and subsequent binding of GFI1 to epigenetically repress *Runx2* in MM-exposed MC4 pre-OB following either direct contact (5TGM1) or indirect (trans-wells, MM1.S) co-culture. As neither of these allow separation of the effects of the inhibitor on each cell type, we showed that XRK3F2 prevents *Gfi1* upregulation in MC4 preOB and primary human BM-MSCs treated with MM1.S conditioned media, or TNFα alone and in combination with IL7. Using blocking antibodies, we previously reported that MM cell down-regulation of *Runx2* mRNA in MC4 preOB cells required both TNFα and IL7 (30). While p62 can transmit signaling from multiple receptor pathways, TNFα signals through the p62-ZZ domain via the TNFα signaling adaptor RIP1 (19). RIP1 binding with the p62-ZZ domain transduces downstream activation of NFκB via the atypical protein kinase Cζ (aPKCζ) (42), as well as transcription factor C/EBPβ via the p38 MAPK pathway (43), which each have binding sites on p62. PKCζ interacts with the N-terminal PB1 domain of p62 and p38 MAPK interacts with the p38 interaction domain, which overlaps the LIM-domain protein binding (LB) region (44, 45). We hypothesize that NFκB and/or C/EBPβ may be involved in transcriptional regulation of *Gfi1* downstream of TNFα receptor (Figure 7). Though *Gfi1* promoter regulation has not yet been well characterized in any cell type, a study by Lidonnici et al. (46) implicated C/EBPα in activation of *Gfi1* in BCR/ABL-expressing cells. Future experiments will delineate the p62-mediated transcription factor regulatory networks regulating *Gif1* activation downstream of cytokine receptor signaling and direct-MM contact in BMSCs.

Together with inhibition of MM-induced *Gfi1* expression, we observed that XRK3F2 increased *Runx2* mRNA in MM-exposed cells and restored osteogenic differentiation, as evidenced by rescued alkaline phosphatase activity and mineralization. This suggested that XRK3F2 treatment enhanced OB differentiation of MM-preOB. We found that XRK3F2 also prevented MM-induced recruitment of GFI1 to the *Runx2* gene, and alleviated its inhibitory chromatin effects (Figure 7). GFI1 interacts with various chromatin remodeling enzymes such as histone deacetylase HDAC1, and histone demethylases G9a and LSD1 to form repressive complexes and target gene promoters (47, 48). Further, we have reported that GFI1 also recruits the transmethylase EZH2, which catalyzes the repressive methylation of H3K27 (31). Our data indicate that XRK3F2 blocked MM-induced GFI1 binding and HDAC1 recruitment to the *Runx2-P1* promoter, thereby preventing MM-induced loss of the transcriptionally permissive chromatin acetylation, H3K9ac at the *Runx2* gene in preOB (Figure 7). It is interesting to note that in the ChIP experiments detecting acetylation levels at *Runx2* (Figure 5B), patients with pre-existing skeletal disease (MM2, 3) responded better to XRK2F2 treatment than the ones without a skeletal disease diagnosis (MM1, 4). Of the mineralization assays in Figure 6A, although they represent a wide variation in their intrinsic differentiation capacity, all three patient BMSC responded to XRK3F2 with increased mineralization; two samples were from MM patients with bone disease and the bone disease status of the third was unknown. Since we demonstrated the importance of targeting the p62-ZZ-GFI1 signaling axis within BMSCs to decrease (or rescue from) their response to MM cells, altogether our patient data suggests that patients with bone involvement may benefit more from XRK3F2 treatment than those without bone disease. Future experiments using additional samples from patients with variety of MM disease stages and skeletal involvement may provide valuable information about the importance of blocking the p62-ZZ-GFI1 signaling axis in MM-BMSC interactions in the clinical setting. Since GFI1 is also subjected to regulation at the level of cytoplasmic vs. nuclear localization (30), we speculate that in addition to transcriptional inhibition, XRK3F2 may also act at the level of post-translational modifications that regulate GFI1 nuclear translocation induced by MM-exposure, TNF, and IL7 signaling in MC4 preOB (Figure 7) (30). In addition, the cytoplasmic shuttling factor LIM domain-containing protein Ajuba has been reported to bind and function as a co-repressor for GFI1, in an Ajuba-GFI1-HDAC protein complex, on select target genes including *Runx2* (49, 50). Interestingly, Ajuba has also been implicated in aPKC/p62 activation of NFκB in response to either TNFα or IL1β in MEFs via binding to the LIM-binding (LB) domain between the ZZ domain and the TRAF6 binding domains (51). Therefore, we hypothesize that XRK3F2 selective blocking of the p62-ZZ domain-signaling module, may also influence cytoplasmic-nuclear shuttling and/or Ajuba-dependent binding of GFI1 to the *Runx2* promoter.

In rescue experiments, in which the myeloma-induced repressive chromatin structure was already established on the *Runx2* gene in preOB before addition of XRK3F2, we found that XRK3F2 can reverse the established epigenetic *Runx2* suppression and alleviate this block to osteogenic differentiation. This is consistent with the results found using XRK3F2 to treat an *in vivo* MM-mouse model in which the tumor was first allowed to grow for 2 weeks before drug administration (29). The clinical implications of this finding are also intriguing as they suggest that MM-induced bone destruction could be reversed, which is particularly important since patients often present with myeloma-induced bone osteolysis at diagnosis (52). In addition to reversing epigenetic suppression of *Runx2* and transcription of several downstream osteogenic genes, XRK3F2 treatment of *ex vivo* expanded primary MM patient BMSCs rescued both epigenetic repression at *Runx2* and osteogenic differentiation reflected in mineralization potential. Since the goal of this study was to understand the mechanism underlying our previously reported work that revealed that XRK3F2 could rescue the bone underlying MM cells in a MM *in vivo* model using 5TGM1 MM cells, we have primarily focused on the use of 5TGM1 and MM1.S myeloma cells in our co-culture experiments. While beyond the scope of this manuscript, due to the heterogeneity associated with MM, future experiments assessing the use of XRK3F2 in the context of additional MM cell lines will be instrumental. This will yield critical information about the requirement of p62 signaling activation within BMSCs triggered by interactions with other subtypes of MM cells.

Despite currently available treatments, the persistence of MM-induced skeletal lesions remains a relevant clinical problem for MM patients. Therefore, a better understanding of the molecular networks involved in sustaining MM-related bone disease is eagerly awaited. While epigenetic mechanisms in BMSCs in the context of MMBD are largely unexplored, here we demonstrate that targeting signal pathways that regulate epigenetic events using the small molecule inhibitor XRK3F2 is of great therapeutic potential, as it exhibits osteo-regenerative properties.

## Author contributions

JA, RS, SM, GR, and DG conceived of the study and designed experiments, analyzed data, and interpreted results. JA, RS, SM, QS, JLA, and DZ performed experiments. RS, X-QX, and GR provided research materials. JA, RS, and DG wrote the manuscript. All contributing authors have agreed to submission of this manuscript for publication.

### Conflict of interest statement

GR serves as a consultant for Amgen. X-QX is the Founder of ID4Pharma and serves as a consultant for Oxis Biotech. The remaining authors declare that the research was conducted in the absence of any commercial or financial relationships that could be construed as a potential conflict of interest.
